# Rapid aging of influenza epidemics in China from 2005/06 to 2016/17: A population-based study

**DOI:** 10.1016/j.idm.2025.02.003

**Published:** 2025-02-04

**Authors:** Weibo Tang, Hao Lei, Nan Zhang, Yaojing Wang, Shimeng Cai, Shuyi Ji, Lei Yang, Mengya Yang, Can Chen, Shigui Yang, Dayan Wang, Yuelong Shu, RIDPHE Group

**Affiliations:** a School of Public Health, The Second Affiliated Hospital, Zhejiang University School of Medicine, Hangzhou, Zhejiang, PR China; b Beijing Key Laboratory of Green Built Environment and Energy Efficient Technology, Beijing University of Technology, Beijing, PR China; c School of Economics, Peking University, Beijing, PR China; d National Institute for Viral Disease Control and Prevention, Chinese Center for Disease Control and Prevention, Key Laboratory for Medical Virology, National Health Commission, Beijing, 102206, PR China; e Key Laboratory of Pathogen Infection Prevention and Control (MOE), State Key Laboratory of Respiratory Health and Multimorbidity, National Institute of Pathogen Biology, Chinese Academy of Medical Sciences & Peking Union Medical College, Beijing, 102629, PR China; f School of Public Health (Shenzhen), Sun Yat-sen University, Shenzhen, 518107, PR China

**Keywords:** Aging, Influenza, Surveillance, Influenza-like illness

## Abstract

**Background:**

China is an aging society, and the older population is at a higher risk of influenza infection and influenza-related mortality. However, there is limited knowledge regarding the aging of influenza epidemics, which is crucial for estimating the disease burden.

**Methods:**

We collected weekly influenza surveillance data from 2005/06 to 2016/17, and quantified the aging of influenza-like illness (ILI) and influenza virus-positive cases in China via the mean age of the influenza cases and the proportion of individuals aged 65 and above among the influenza cases.

**Results:**

On average, the mean age of ILI cases and influenza-positive cases increased by 0.52 years and 0.60 years per year, respectively, which is approximately three times the annual increase in the mean age of the population. Additionally, the proportion of individuals aged 65 and above among influenza-positive cases increased from 0.5% to 4.0%. The aging of patients infected with influenza B/Yamagata was the most rapid, with a mean age increase of 0.73 years per year, followed by those infected with influenza A (H1N1) and influenza A (H3N2). Conversely, there was no significant increase in the mean age of patients infected with influenza B/Victoria. The aging rate of influenza epidemics in southern China was significantly higher than in northern China.

**Conclusions:**

Based on estimates of excess mortality due to influenza from 2010/11 to 2014/15, by 2050, the annual number of respiratory disease-related deaths associated with influenza is projected to increase 2.5-fold. This finding highlights the importance of influenza vaccination among older individuals in China.

## Highlights

• The aging of influenza epidemics is crucial for estimating the disease burden.
• The mean age of ILI cases and influenza-positive cases increased by 0.52 years and 0.60 years per year, respectively.
• By 2050, the annual number of influenza-related deaths is projected to increase 2.5-fold in China.

## 1 Background

The increase in life expectancy and sustained low fertility rates have led to an aging population worldwide. China is one of the countries experiencing the fastest aging process, with a larger population of individuals aged 65 and above than any other country in the world (Lancet, 2016). According to China's seventh national census of 2020, the population of older individuals aged 65 and above was 190.64 million, accounting for 13.50% of the total population (National Bureau of Statistics of the People's Republic of China, 2021). The increasing burden of noncommunicable diseases accompanying population aging has received much attention (Chaker et al., 2015; Kämpfen et al., 2018; Wang & Chen, 2022). However, the increasing burden of infectious diseases, particularly influenza epidemics in an aging population, is less well understood. The older population faces a higher risk of influenza (Lei et al., 2020), because immunosenescence enhances susceptibility to influenza viruses and reduces vaccine effectiveness (Hernandez-Vargas et al., 2014). In addition, influenza-attributable or excess mortality exponentially increases with age (Thompson et al., 2003). In China, it is estimated that approximately 80% of influenza-associated excess respiratory deaths occur in people aged 60 years or older (Li et al., 2019). Thus, quantifying the aging of influenza cases has become crucial in evaluating the disease burden of influenza and optimizing vaccination strategies.

Modelling studies have used mathematical models to evaluate the impact of population aging on the burden of influenza in high-income countries. For example, in the US, as the population ages, the estimated influenza cases are projected to increase by 26%, and annual influenza direct medical costs are expected to increase by 49% from 2017 to 2047 (Talbird et al., 2021). In the Netherlands, assuming unchanged incidence rates of influenza over time, the total influenza-associated disability-adjusted life years are predicted to increase by 2.3-fold over a 30-year period in the aging population (McDonald et al., 2012). These modelling studies have predicted an increasing disease burden with population aging. In high-income countries, urban living has become the norm in the last century. Therefore, other factors, such as contact heterogeneity among different age groups (Lei et al., 2020), may have a limited impact on influenza transmission dynamics. However, in developing countries with rapid urbanization over the past decades, such as China, these factors may exacerbate the aging of influenza cases.

This study aimed to estimate the aging of influenza epidemics by utilizing influenza surveillance data from 2005 to 2017, made possible by the availability of extensive influenza surveillance data in China.

## 2 Methods

### 2.1 Data sources

In this study, we used weekly reports of influenza surveillance data provided by the Chinese National Influenza Center. The influenza surveillance data were based on specimens tested at 556 sentinel hospitals and 411 network laboratories located across 31 provinces of Mainland China (including autonomous regions and municipalities) (Shu et al., 2019). The collection of high-quality influenza surveillance data in China commenced in 2005 (Shu et al., 2019). Each week, sentinel hospitals report the number of outpatient visits and influenza-like illnesses (ILI). ILI was defined using a standard case definition: body temperature ≥ 38° C with either cough or sore throat, in the absence of an alternative diagnosis (Xu et al., 2013). Additionally, a convenience sample of patients visiting sentinel hospitals within 3 d of ILI onset was collected, with 5–15 nasopharyngeal swab samples taken each week (Xu et al., 2013). Demographic and epidemiologic data, including age, sex, date of illness onset, and occupation, were also collected. Patient specimens were tested using real-time reverse transcription polymerase chain reaction or virus isolation in affiliated laboratories. The aging of the ILI cases and influenza positive cases was evaluated respectively.

### 2.2 Statistical analysis

Based on previously observed influenza seasonality patterns in China (Lei et al., 2022), we defined the epidemiological annual cycle of influenza activity as the period from week 14 to week 13 of the following year. Therefore, we considered the 2005/06 epidemiological cycle to span from week 14 in 2005 to week 13 in 2006. During the study period from 2005/06 to 2016/17, significant enhancements and expansions were implemented within the influenza surveillance network following the 2009 influenza A/H1N1 pandemic (Shu et al., 2019). Subsequently, there was a substantial increase in the number of specimens tested after the epidemiological cycle of 2009/10 (Shu et al., 2019). To examine the robustness of the observed aging of influenza cases in China, we conducted separate analyses of province-level influenza surveillance data before and after the 2009 influenza A/H1N1 pandemic. In addition, given the vast geographical expansion of China and variations in the population age distribution among different provinces (Lei et al., 2023), this study aimed to quantify the extent of aging in influenza cases relative to population aging. To achieve this, we performed a thorough analysis of the province-level influenza surveillance data. After excluding Tibet due to incomplete influenza surveillance data, our analysis included data from the 30 provinces in China.

When comparing the aging of population and influenza epidemics in China, following the age grouping in the China Statistical Yearbook, (National Bureau of Statistics of China) the continuous age range was categorized into three age groups: ≤ 14 years, 15–64 years, and ≥ 65 years. A two-sided Kolmogorov–Smirnov (K-S) test was used to access the normal distribution, The K-S test compares the empirical cumulative distribution function (CDF) of the sample data with the CDF of a reference normal distribution. The K-S test statistic *D* is defined as:

D=max|Fn(x)−F(x)|

Where *D* is the Kolmogorov-Smirnov statistic, representing the maximum absolute difference between the empirical CDF *Fn*(*x*) and the theoretical CDF *F*(*x*). *Fn*(*x*) is the empirical CDF of the sample. *F*(*x*) is the CDF of the reference normal distribution. The test returns the *D* statistic and a corresponding *p*-value. If the *p*-value is smaller than0.05, the null hypothesis that the data follows a normal distribution is rejected.

To quantify the aging speed of influenza epidemics and the population, linear regression between the aging of influenza cases and aging of population in China ware performed. The regression model is expressed as:

$$Y=\beta_{0}+\beta X+\epsilon$$

Where Y is the proportion of a certain age group in influenza cases, while X is the proportion of a certain age group in the population. $\beta_{0}$ is the intercept and ϵ is the error. The coefficients β is estimated using the method of least squares, which minimizes the sum of squared residuals:

$$\min\sum_{i=1}^{n}{\left(Y_{i}-{\hat{Y}}_{i}\right)}^{2}$$

The goodness of fit of the regression model is assessed using the coefficient of determination *R*^2^, which is calculated as:

$$R^{2}=1-\frac{SS_{res}}{SS_{tot}}$$

Where SS_res_ is the sum of squared residuals, SS_tot_ is the total sum of squares. An $R^{2}$ value closer to 1 indicates that the independent variables explain a large proportion of the variance in the dependent variable, while a value closer to 0 suggests a weaker explanatory power. For linear regression, the *p*-value for each regression coefficient is estimated by assessing the likelihood that the observed relationship between the independent variable and the dependent variable happened by chance. The *p*-value is derived from the t-statistic, which is calculated as:

$$t=\frac{\beta }{SE\left(\beta \right)}$$

Where β is the estimated coefficient, SE (β) is the standard error of the coefficient. When *p* < 0.05, it indicates that the linear relationship between two corresponding variables is statistically significant. All analyses were performed using ***R*** software Version 4.3.1 (R Core Team (2023). R: A Language and Environment for Statistical Computing. R Foundation for Statistical Computing, Vienna, Austria. https://www.R-project.org/.)

## 3 Results

### 3.1 Influenza specimen testing and positivity

During the study period, from week 14, 2005, to week 13, 2017, a total of 3,682,614 ILI cases were tested, and 505,533 were positive for the influenza virus, yielding an influenza virus-positive rate of 13.7%. Of the patients with influenza, 54.7% were male (Table 1). Among the ILI cases, 64.4% were children under the age of 18 years, and 63.7% of the positive cases were also in children under 18 years of age (Table 1). Approximately two-thirds of the influenza surveillance data were obtained from southern China (Table 1).

**Table 1:** Characteristics of influenza cases in mainland China, 2005/06 through 2016/17.

Variables	Level	ILI cases (%)	Positive cases (%)
Sex	Male	2013141 (54.7%)	276502 (54.7%)
	Female	1669473 (45.3%)	229031 (45.3%)
Age group	=< 14	2222996 (60.4%)	284510 (56.3%)
	15–59	1331212 (36.1%)	206243 (40.8%)
	≥ 60	128406 (3.5%)	14780 (2.9%)
Region	Southern China^a^	2316404 (62.9%)	323549 (64.0%)
	Northern China	1352293 (36.7%)	180133 (35.6%)

a Except Tibet; ILI: influenza-like illnesses.

The positivity rate for the influenza virus exhibited considerable variability, ranging from 9.26% in 2016/17 to 35.2% in 2009/10 (Fig. S1). The peak positivity in 2009/10 was mainly due to the 2009 influenza A/H1N1 pandemic. The annual number of specimens tested was fewer than 100,000 before the 2009 influenza A/H1N1 pandemic. After the surveillance network was improved and expanded following the 2009 influenza A/H1N1 pandemic, the annual number of specimens tested increased substantially to more than 200,000, and after the 2012/13 epidemiological year, this number further rose to approximately 500,000. Since the 2009/10 epidemiological year, the positivity rate for the influenza virus has remained stable at approximately 10% (Fig. S1).

### 3.2 Age of influenza cases

**Fig. 1:**
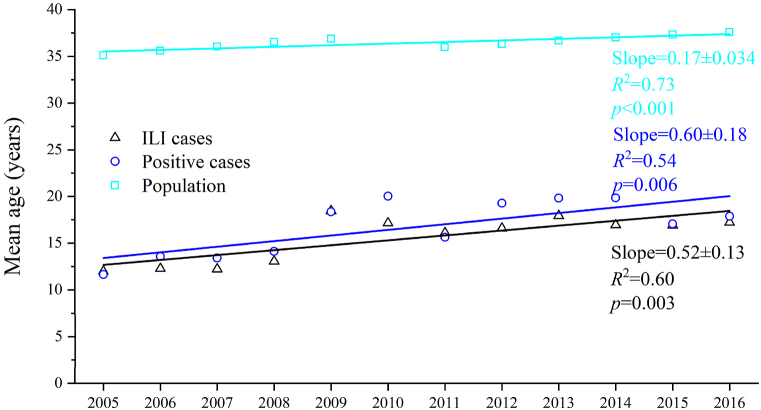
Mean age of the ILI cases with specimens collected and those tested positive for influenza, China, 2005/06–2016/17. ILI: influenza-like illnesses.

**Fig. 2:**
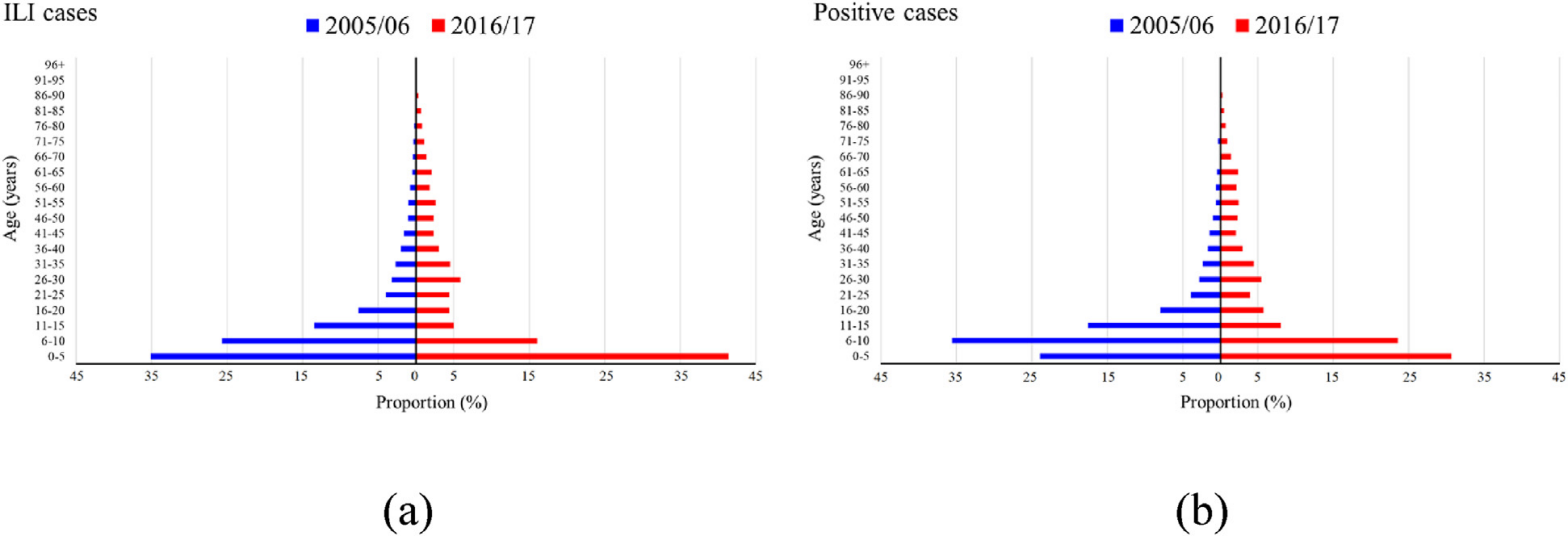
Age distribution of (a) ILI cases and (b) influenza-positive cases in China in the epidemiological years 2005/06 and 2016/17, respectively. ILI: influenza-like illnesses.

The mean age of ILI cases subjected to specimen testing showed an upward trend, rising from 12.0 years in 2005/06 to 17.2 years in 2016/17. Similarly, the mean age of influenza-positive cases increased from 11.6 years in 2005/06 to 17.9 years in 2016/17 (Fig. 1). On average, the mean age of ILI cases and influenza-positive cases increased by 0.52 years and 0.60 years per year, respectively, which was much faster than the aging of the population (0.17 year per year; Fig. 1). Notably, during 2009/10 and 2010/11, there was a substantial increase in the mean age of both ILI and influenza-positive cases. When excluding the epidemiological years of 2009/10 and 2010/11 from the analysis, the mean age of ILI cases and influenza-positive cases still exhibited an annual increase of 0.58 years and 0.65 years, respectively (Fig. S2). Stratified analysis of positive-case data revealed that, with the exception of B∖Victoria, the mean age of cases for the other three influenza virus subtypes/lineages increased. Specifically, the mean age of B∖Yamagata cases demonstrated the most rapid increase at 0.73 years per year, followed by A (H1N1) and A (H3N2), which showed annual increases of 0.58 years and 0.45 years, respectively. Conversely, the mean age of B/Victoria cases remained relatively young, showing no significant temporal trend (*p* = 0.106) (Fig. S3). The detailed age distributions of ILI and influenza-positive cases for the epidemiological years 2005/06 and 2016/17 are shown in Fig. 2. The age distribution of ILI and influenza-positive cases deviated from the normal distribution (*p* < 0.05, two-sided Kolmogorov–Smirnov test). Compared with the epidemiological year 2005/06, in the epidemiological year 2016/17, although children under 10 years of age still comprised more than half of the influenza cases, there was a higher prevalence of ILI and influenza-positive cases among older individuals (Fig. 2a and b). Further analysis examined the detailed age distribution of cases across the four influenza virus subtypes for the epidemiological years of 2005/06 and 2016/17. These findings are consistent with those derived from the overall data of the positive cases (Fig. S4).

**Fig. 3:**
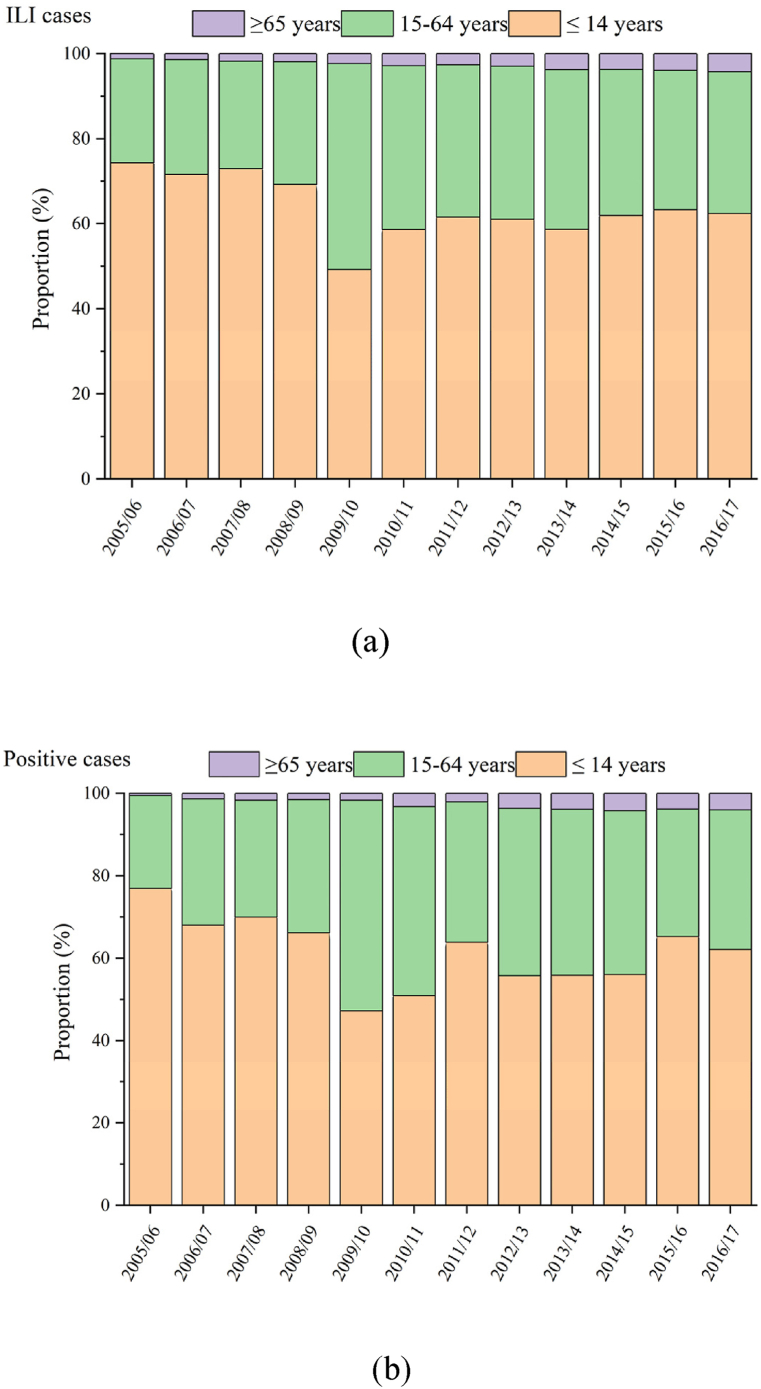
Proportion of three age groups: (1) children ≤ 14 years, (2) younger adults aged 15–64 years, and (3) older adults ≥ 65 years. (a) ILI cases with specimens collected, and (b) ILI cases tested positive for influenza, China, 2005/06–2016/17. ILI: influenza-like illnesses.

During the study period from 2005/06 to 2016/17, the proportion of ILI cases among older adults aged >64 years increased from 1.1% to 4.2% (Fig. 3a), and the proportion of influenza-positive cases increased from 0.5% to 4.0% (Fig. 3b). Furthermore, there was an increase in the proportion of younger adults (aged 15–64 years) in both the ILI and influenza-positive cases (Fig. 3). The proportion of children under 15 years of age decreased from 74.3% to 62.4% in ILI cases and from 77.0% to 62.2% in influenza-positive cases (Fig. 3). Additionally, during the study period, the proportion of older adults increased among the cases involving all three influenza virus subtypes, except B/Victoria (Fig. S5).

**Fig. 4:**
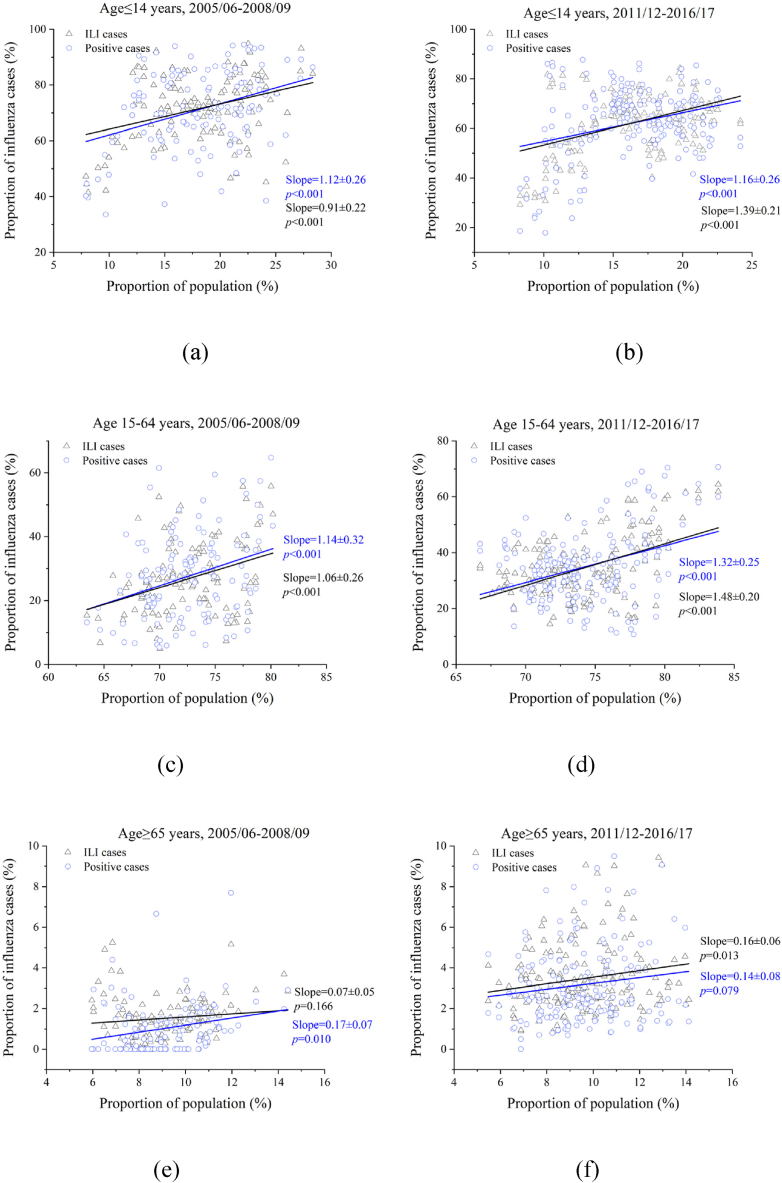
Proportion of three age groups in influenza cases compared with those in the population before and after the 2009 influenza A/H1N1 pandemic, respectively. (a and b) children ≤ 14 years; (c and d) younger adults 15–64 years; (e and f) older adults ≥ 65 years. (a, c, and e) during 2005/06–2008/09; (b, d, and f) 2011/12–2016/17. ILI: influenza-like illnesses.

The changes in influenza cases in the three age groups compared with those in the Chinese population before and after the 2009 influenza A/H1N1 pandemic are presented in Fig. 4. Generally, the aging rate of influenza cases after the 2009 influenza A/H1N1 pandemic was slightly higher than that before the pandemic. On average, a 1% decrease in the population of children under 15 years of age correlated with a decline of 0.91–1.12% in the proportion of children with influenza before the pandemic (Fig. 4a) and a decline of 1.16–1.39% after the pandemic (Fig. 4b). Similarly, a 1% increase in the proportion of younger adults led to an increase of 1.06–1.14% and 1.32–1.48% in the proportion of younger adults among influenza cases before and after the pandemic, respectively (Fig. 4c and d). There was no statistically significant (*p* < 0.01) linear correlation between the proportion of older adults in the population and influenza cases (*p* > 0.01) (Fig. 4e and f). The main reason for this could be that the proportion of older adults aged >65 years still accounted for a very small percentage of influenza cases; thus, the randomness of specimen collection played an important role. The different rates of change observed among the three age groups for influenza cases and populations suggest that factors beyond population aging may contribute to the aging of influenza cases.

**Fig. 5:**
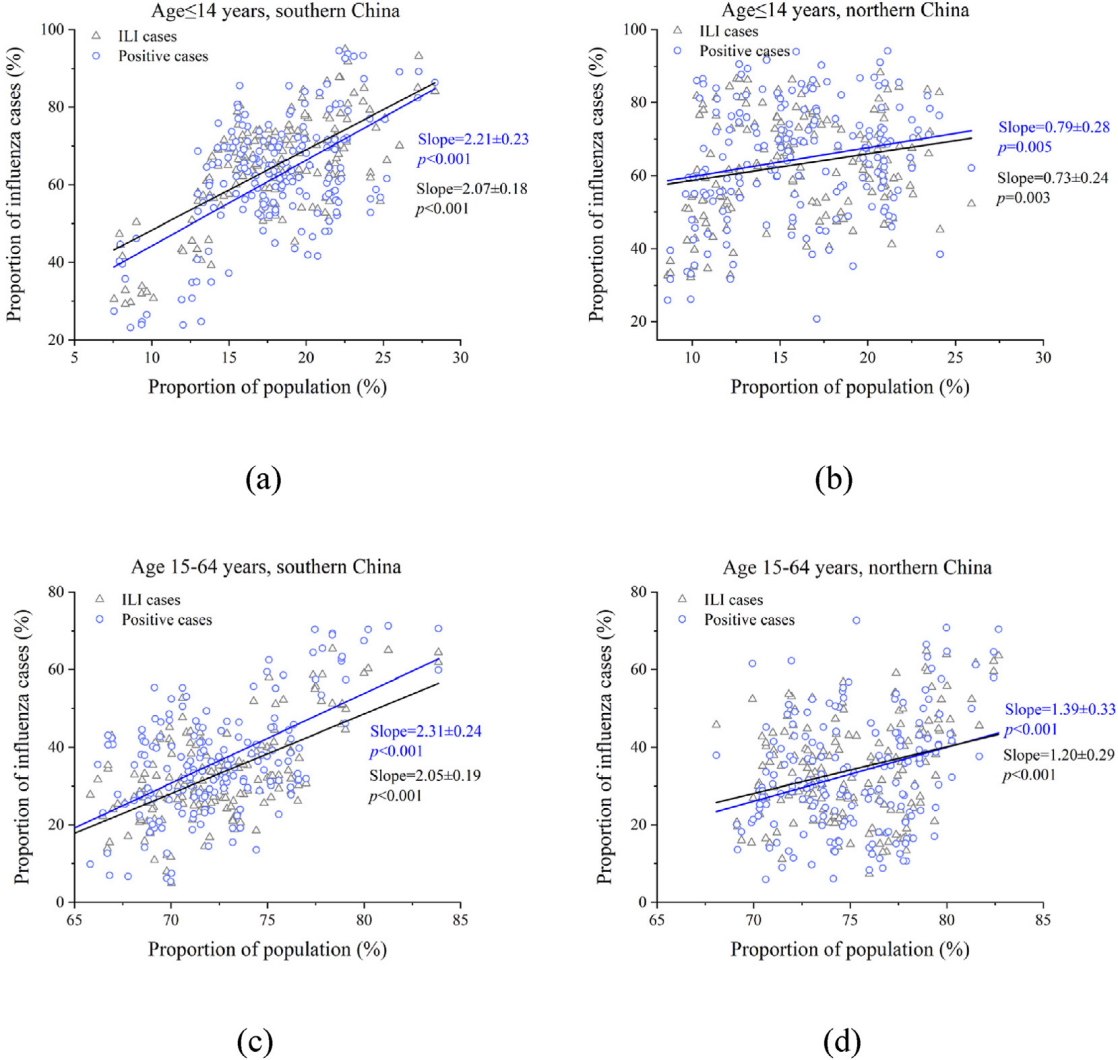
Proportion of children ≤ 14 years and younger adults 15–64 years in influenza cases compared with those in the population in southern and northern China, respectively. (a and b) children ≤ 14 years; (c and d) younger adults 15–64 years; (a and c) in southern China; (b and d) in northern China. ILI: influenza-like illnesses.

The aging of influenza epidemics in southern China was much faster than that in northern China (Fig. 5). On average, a 1% decrease in the population of children under 15 years of age led to a decline of 2.07–2.21% in the proportion of children among influenza cases in southern China and only a 0.73–0.79% decline in northern China (Fig. 5a and b). For younger adults, a 1% increase in the population led to 2.31% and 1.39% increases in influenza-positive cases in southern and northern China, respectively (Fig. 5c and d).

## 4 Discussion

In China, there is an annual average of 88,100 influenza-associated excess respiratory deaths (Li et al., 2019). Since 2000, China has entered a phase of population aging, and this rapid aging trend continues (Fang et al., 2015; Han et al., 2020). Given the current demographic trends, population aging has become a significant factor influencing health policy and disease prevention strategies. Thus, exploring the aging of influenza cases is important for estimating the disease burden of influenza in China. The influenza-associated excess mortality rate for individuals aged 60 years or older is 26 times higher than that among those under 60 years (Li et al., 2019, 2023). The influenza epidemic in the context of China's rapidly aging population raises substantial concerns if proper interventions are not implemented. The estimated excess influenza-associated respiratory mortality rates in China from 2010/11 through 2014/15 were 1.5 per 100,000 person-seasons for individuals younger than 60 years and 38.5 per 100,000 person-seasons for individuals aged 60 years or older (Li et al., 2019). By 2050, the percentage of older persons aged 60 years or older is projected to increase to 38.81% (Department of Economic and Social AffairsPopulation and Division, 2022). Assuming the total population remains at 1.4126 billion, the estimated annual number of influenza-associated excess respiratory deaths would increase to 224,000, without considering the impact of influenza virus evolution and climate. This estimate is roughly 2.5 times higher than the recent annual influenza-associated excess respiratory deaths (Li et al., 2019). This increase is much higher than the 26% increase in influenza cases projected in the US (Talbird et al., 2021), but close to the 2.3-fold increase in total influenza-associated disability-adjusted life years in the Netherlands (McDonald et al., 2012) over a 30-year period of an aging population. In addition, the aging of influenza cases leads to an increased economic burden, as the severity of influenza-associated cases, hospitalization rates, and cost per hospitalization all increase with age (Andrew et al., 2019; Yang et al., 2015). These highlight the urgent need for tailored public health interventions, particularly those focused on older adults who bear the brunt of influenza-related mortality.

Recently, adults aged 64 years and older have accounted for approximately 11% of the population in China, yet they represent only approximately 4% of influenza cases. This could be primarily attributed to the fact that older adults in this age group have the lowest mean number of contacts (Mossong et al., 2008; Zhang et al., 2020). However, the lower rate of recorded cases in older adults does not necessarily indicate a lower disease burden, as older individuals are more likely to suffer from severe outcomes or complications when they are infected by influenza. By contrast, children under the age of 15 years, who made up approximately 18% of the population, account for over 60% of influenza cases. This is primarily because of their higher susceptibility to the influenza virus and the high rate of contact among children in schools, which facilitates the spread of the virus (Cauchemez et al., 2009; Mossong et al., 2008). Our previous simulation studies indicated that the age distribution of influenza cases in China exhibits a similar trend (Lei et al., 2023, 2024). Our research shows that with the ongoing aging of the population in China, the aging of influenza cases is noticeable, contributing to an increasing burden of influenza. Stratified analysis based on influenza virus subtypes/lineages revealed that the aging progress of influenza epidemics varied among the different subtypes/lineages (Fig. S3). Prioritizing control measures for influenza virus subtypes/lineages that exhibit faster aging rates could offer a novel approach for mitigating the influenza burden. After the COVID-19 pandemic, the B/Yamagata subtype become extinct worldwide (Paget et al., 2022). This may mean the decrease of the influenza cases in the elderly since the mean age of the cases infected by B/Victoria is much smaller that these infected by B/Yamagata (Fig. S3). In addition, the rate of change varied among the three age groups, suggesting that factors other than population aging, such as patterns of interpersonal contact, may exacerbate the aging of patients with influenza. Population aging is associated with economic development and urbanization in China. With urbanization, the contact patterns between individuals change, and these changes vary among different age groups. The mean contact rate among school-aged children has not change significantly, as class sizes have not changed substantially (Lei et al., 2023, 2024). By contrast, contact rates among younger adults may change significantly, as population density in urban areas increases exponentially with urbanization (Lei et al., 2023, 2024). The relatively slow increase in influenza cases among older adults might be due to the decreased household size with urbanization (Lei et al., 2023), as the household may be a major indoor environment for influenza transmission to older individuals. Furthermore, with urbanization, the effectiveness of school closures in mitigating seasonal influenza outbreaks has diminished (Lei et al., 2021). This observation provides additional support for the aging phenomenon of influenza cases in China. The aging rate of influenza cases in southern China is much higher than that in northern China. This may be primarily attributed to better economic development and faster urbanization in southern China than in northern China during the past decades. (National Bureau of Statistics of China) Better economic development leads to more contact between people, especially among younger adults, thus exacerbating the aging of influenza cases.

Our study has a major limitation that we were unable to account for the changing surveillance efforts among different age groups, which may introduce observational bias. It is likely that children, who are more prone to seeking medical care due to parental concerns and the severity of symptoms (Switzer et al., 2022), are overrepresented in the surveillance data. As shown in Fig. 3, the percentage of children among influenza cases is disproportionately higher than their proportion in the general population. This suggests that the incidence and risk of illness in adults might be underestimated within the current surveillance system. The differential healthcare-seeking behaviors across age groups could skew the observed age distribution of influenza cases, potentially impacting the accuracy of our findings.

## 5 Conclusions

Studies have suggested prioritizing influenza vaccination for older people (Yang et al., 2015). The aging of influenza cases further highlights the importance of the influenza vaccine for older individuals in China, particularly given that vaccination coverage among older adults in China has been at a low level compared with that in Europe and the USA (Zhang et al., 2023a). Now the influenza vaccination rate was only 2.47%–3.16% in China (Zhao et al., 2022). And only a few provinces in China, such as Beijing and Zhejiang, provided free trivalent influenza vaccines to old adults (Zhang et al., 2023b; Zhao et al., 2022). Though the free influenza vaccination police could raise the influenza vaccination rates to 31.5%–51.8% among high-risk people (Lei et al., 2025; Zhao et al., 2022), still much lower than the 75% targeted influenza vaccination coverage from WHO. All these enhance the importance of influenza vaccination in the elderly people.

## CRediT authorship contribution statement

**Weibo Tang:** Writing – original draft, Visualization, Software, Formal analysis, Data curation. **Hao Lei:** Writing – review & editing, Writing – original draft, Project administration, Methodology, Investigation, Formal analysis, Data curation, Conceptualization. **Nan Zhang:** Writing – review & editing, Data curation. **Yaojing Wang:** Writing – review & editing. **Shimeng Cai:** Data curation. **Shuyi Ji:** Data curation. **Lei Yang:** Data curation. **Mengya Yang:** Data curation. **Can Chen:** Data curation. **Shigui Yang:** Writing – review & editing, Supervision, Conceptualization. **Dayan Wang:** Supervision, Data curation, Conceptualization. **Yuelong Shu:** Writing – review & editing, Supervision, Data curation.

## Ethics approval

Not applicable.

## Availability of data and materials

Due to potentially sensitive information included, the original dataset is not made public and is available from the corresponding author upon reasonable request.

## Funding

This study was funded by grants the 10.13039/501100001809National Natural Science Foundation of China (Grant No. 82003509 to H.L.), 10.13039/501100012166National Key Research and Development Program of China (Grant No. 2021YFC2300100 to Yuelong Shu.).

## Declaration of competing interest

The authors declare that they have no known competing financial interests or personal relationships that could have appeared to influence the work reported in this paper.
