# Supplementary material for: Rapid aging of influenza epidemics in China from 2005/06 to 2016/17: A population-based study
Source: Infect Dis Model. 2025 Feb 4;10(2):639–48. doi: 10.1016/j.idm.2025.02.003 (PMC11869495; doi:10.1016/j.idm.2025.02.003)
Supplement: Multimedia component 1 [file mmc1.docx]

**Rapid aging of influenza epidemics in China from 2005/06 to 2016/17: a population-based study**

**Supplementary material**

**
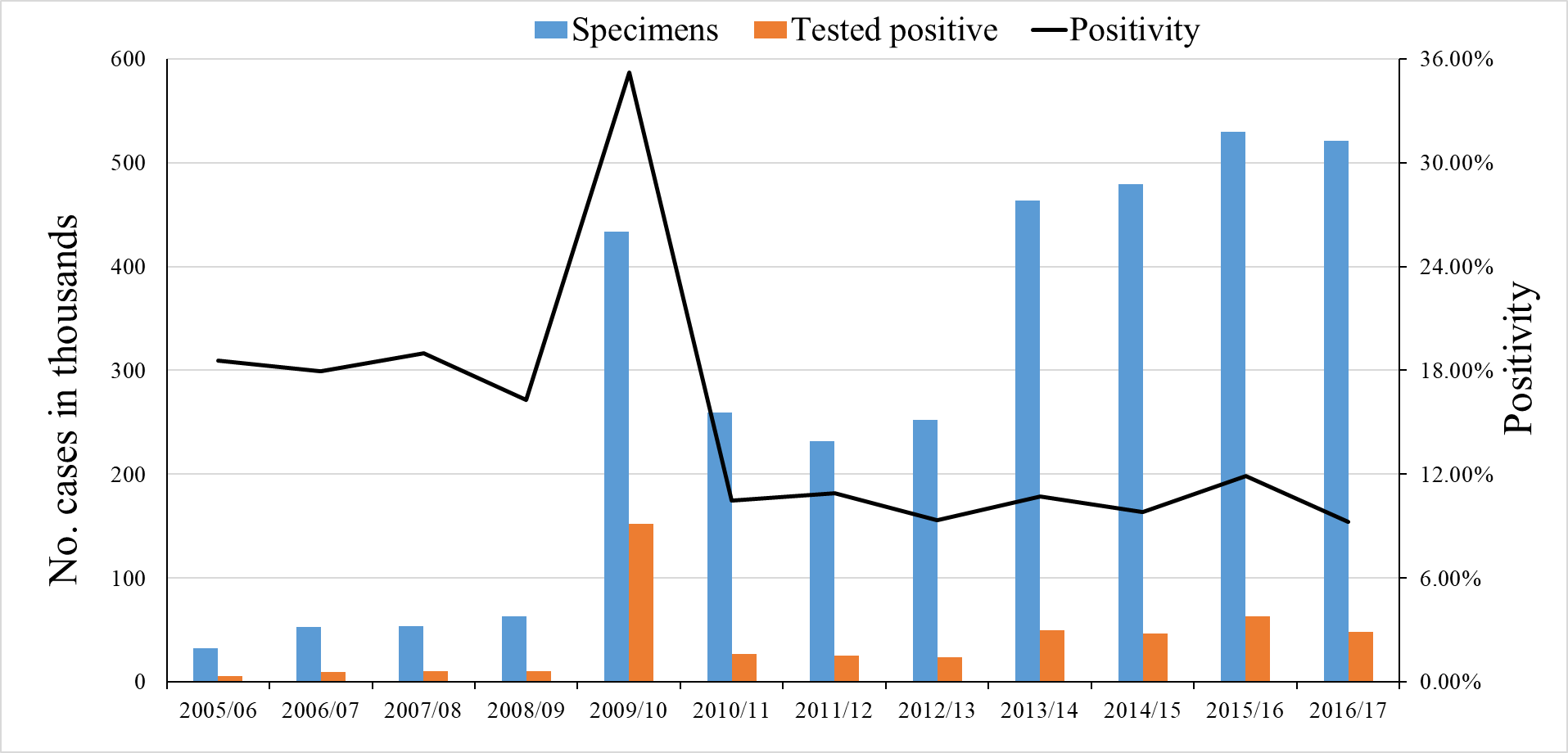
**

**Figure S1**. Number of respiratory specimens collected, tested positive for influenza, and influenza virus positivity from influenza surveillance network, China, 2005–2017.


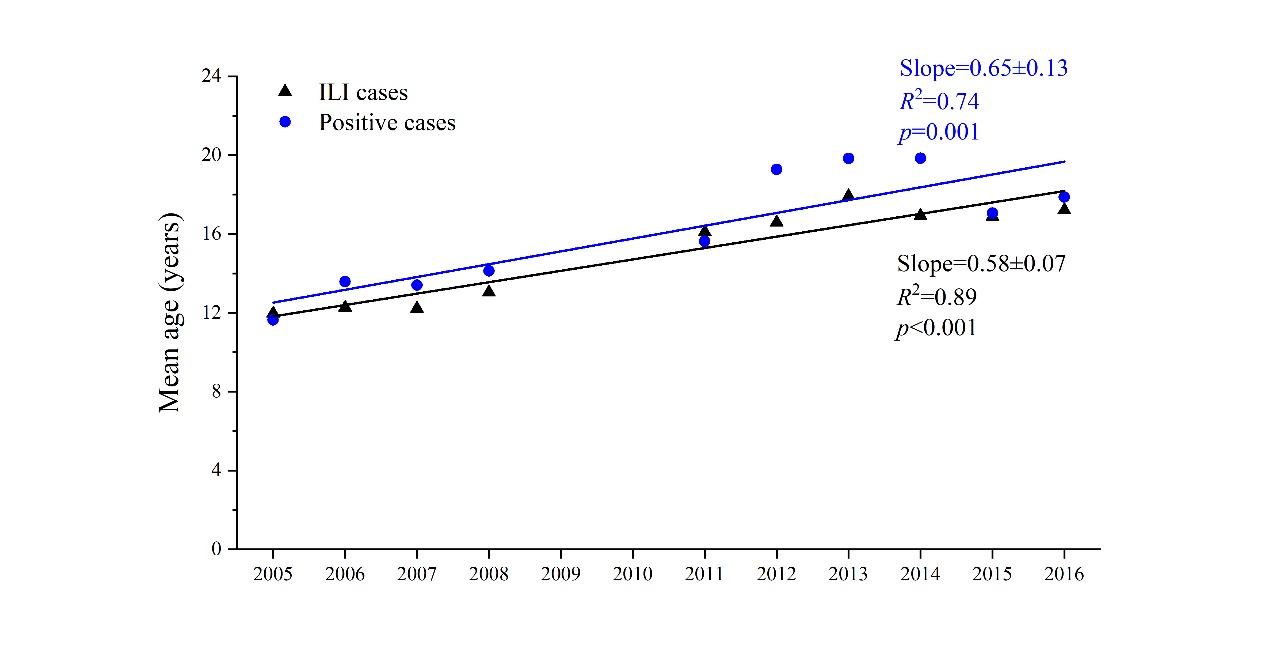


**Figure S2**. Mean age of the ILI case with specimens collected and theses tested positive for influenza, China 2005/06–2016/17, except the epidemiological year 2009/10 and 2010/11 due to the 2009 influenza A/H1N1 pandemic. ILI：influenza like illness.


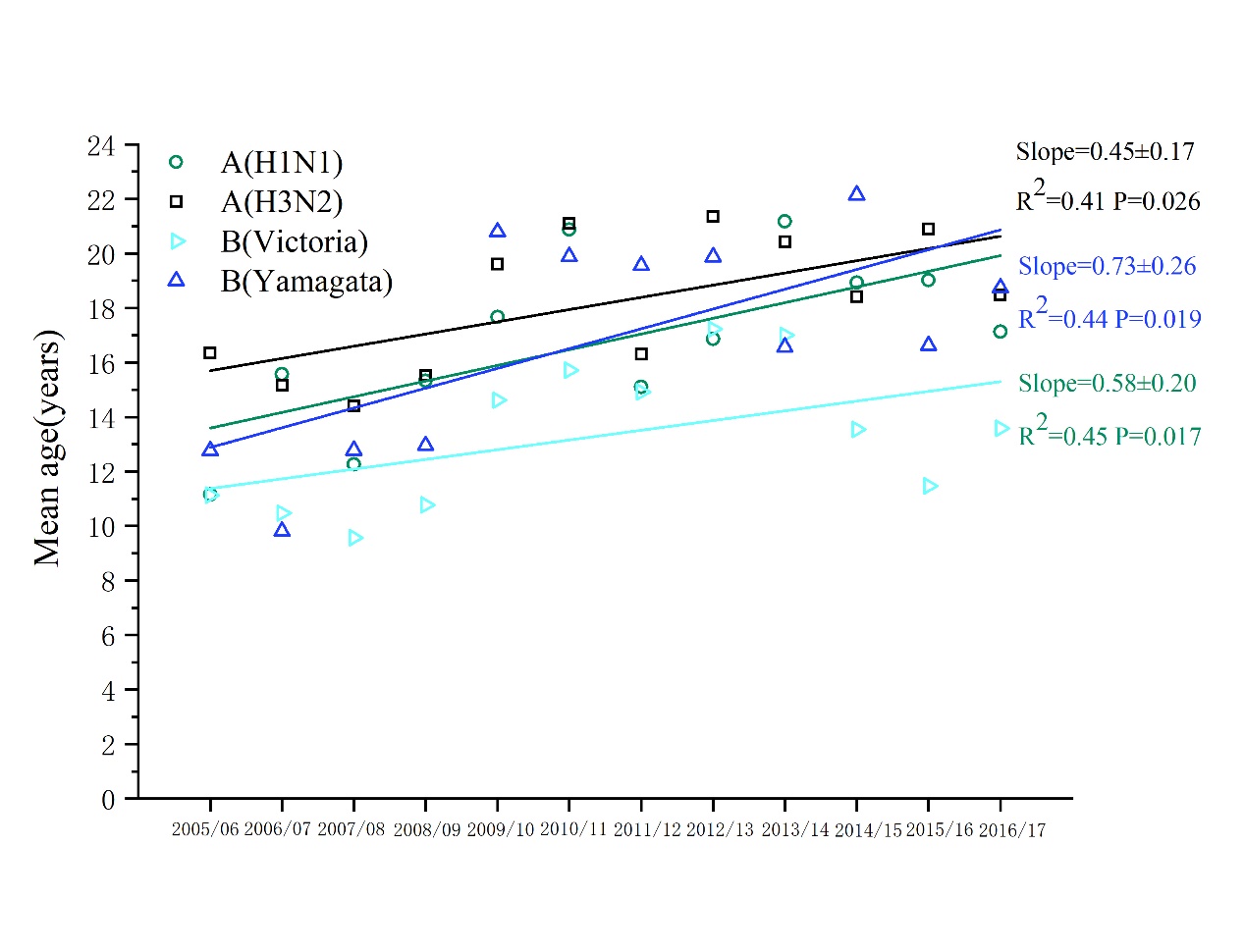


**Figure S3**. Mean age of positive cases with four influenza virus subtypes/lineages, China 2005/06–2016/17.


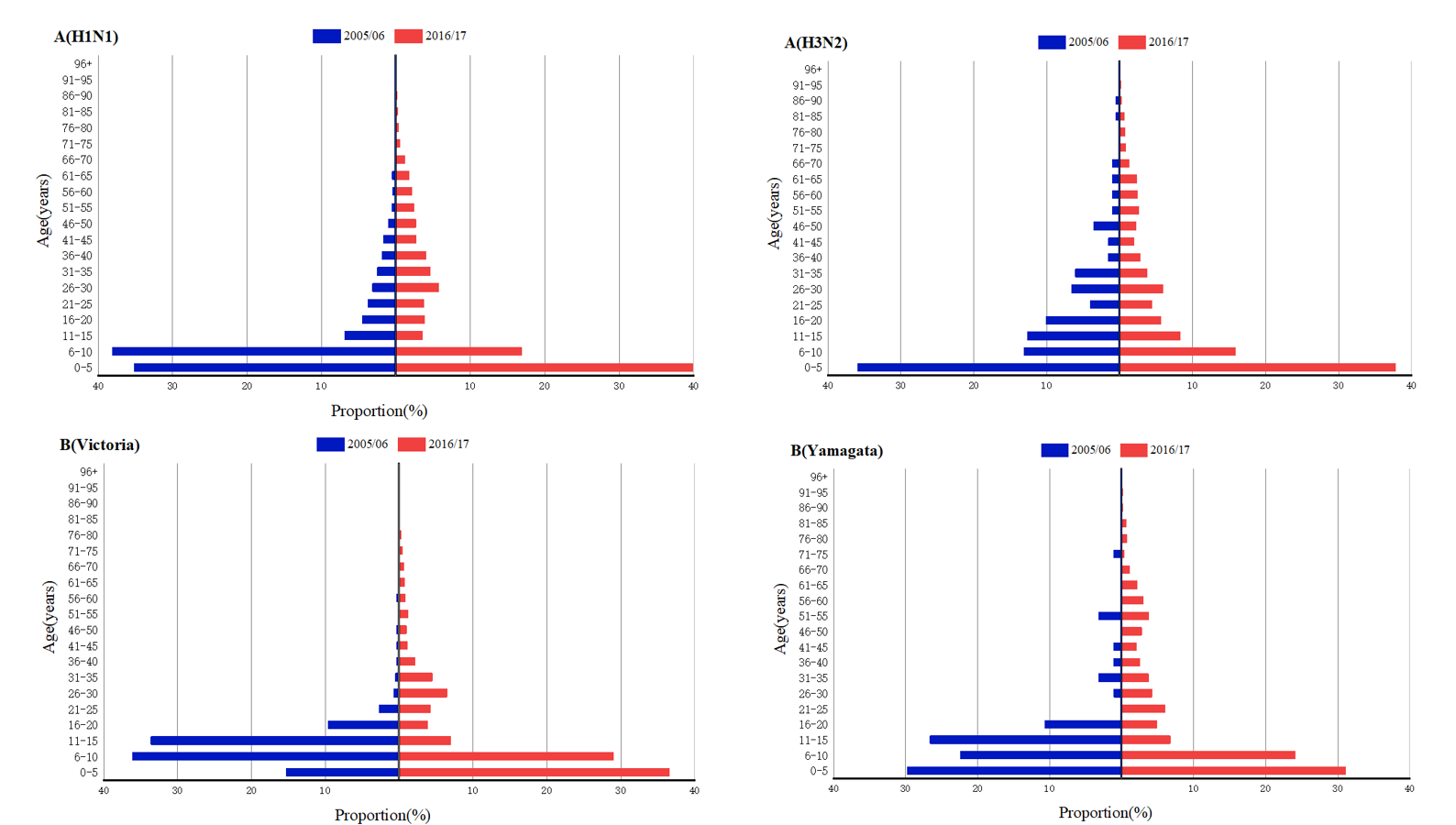


**Figure S4**. Age distribution of positive cases with four influenza virus subtypes/lineages, China 2005/06–2016/17.


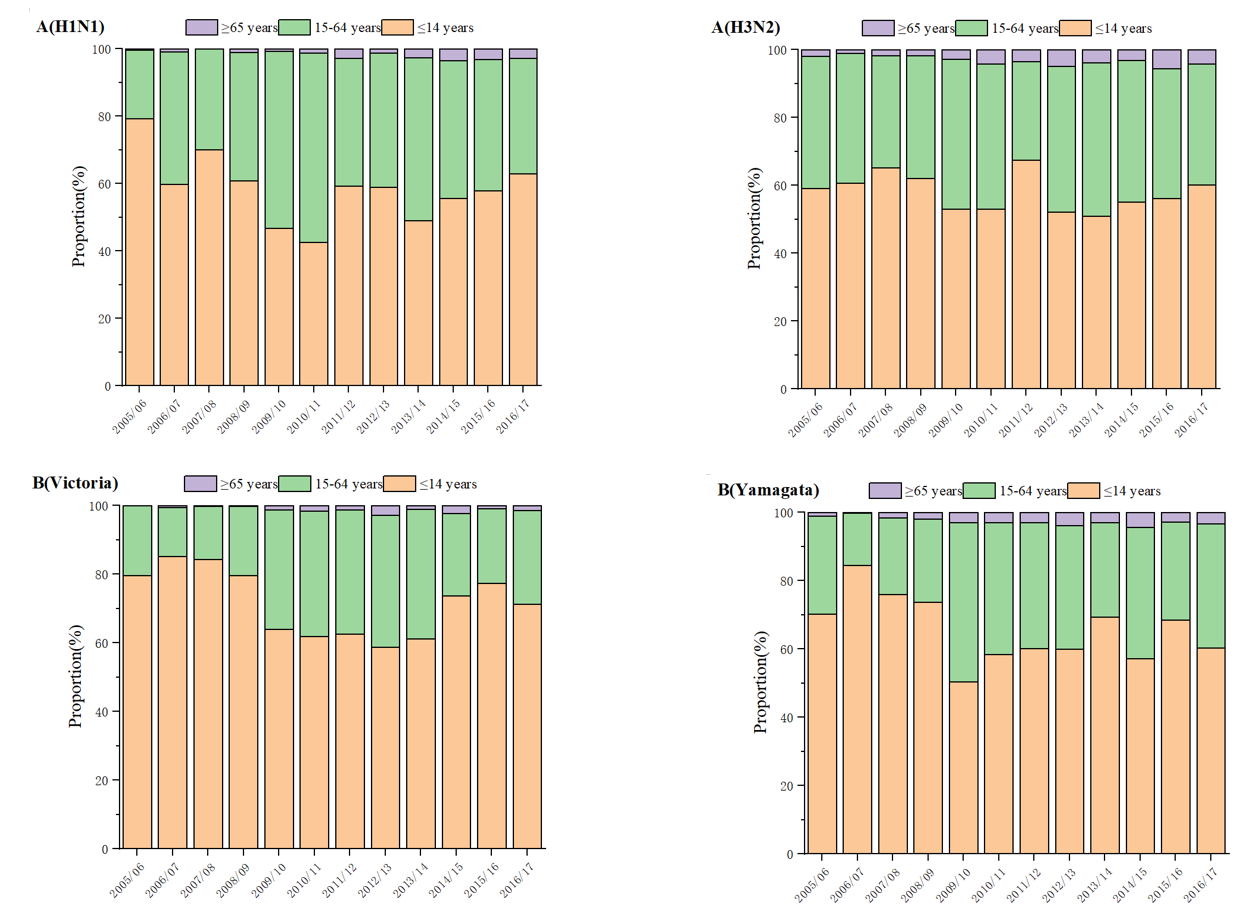


**Figure S5**. Proportion of three age groups, i.e., (1) children ≤14 years, (2) younger adults aged 15-64 years, (3) older adult ≥65 years. in positive cases with four influenza virus subtypes/lineages, China, 2005/06–2016/17.
